# Quantification of [^11^C]ABP688 Binding to mGluR5 in Human Brain using Cerebellum as Reference Region: Biological Interpretation and Limitations

**DOI:** 10.2174/1570159X23666250127161855

**Published:** 2025-02-18

**Authors:** Michele S. Milella, Luciano Minuzzi, Chawki Benkelfat, Jean-Paul Soucy, Alexandre Kirlow, Esther Schirrmacher, Mark Angle, Jeroen A.J. Verhaeghe, Gassan Massarweh, Andrew J. Reader, Antonio Aliaga, Jose Eduardo Peixoto-Santos, Marie-Christine Guiot, Eliane Kobayashi, Pedro Rosa-Neto, Marco Leyton

**Affiliations:** 1 Toxicology Unit, Policlinico Umberto I Hospital-Sapienza, University of Rome, Rome, Italy;; 2 Department of Psychiatry and Behavioural Neurosciences, McMaster University, Hamilton, ON, Canada;; 3 Department of Neurology and Neurosurgery, Montreal Neurological Institute, McGill University, Montreal, Canada;; 4 Department of Psychiatry, McGill University, Montreal, Canada;; 5 Department of Oncology, Division of Oncological Imaging, University of Alberta, Edmonton, AB, Canada;; 6 Molecular Imaging Center Antwerp (MICA), University of Antwerp, Antwerp, Belgium;; 7 Department of Biomedical Engineering, King's College, London, United Kingdom;; 8 Department of Neurology and Neurosurgery, Paulista Medical School, UNIFESP, Sao Paulo, Brazil;; 9 Department of Pathology, McGill University, Montreal, Canada;; 10 Translational Neuroimaging Laboratory, Douglas Research Institute, McGill University, Montreal, Canada

**Keywords:** [^11^C]ABP688, autoradiography, kinetic modeling, mGluR5, positron emission tomography, receptor imaging

## Abstract

**Introduction:**

*In vitro* data from primates provide conflicting evidence about the suitability of the cerebellum as a reference region for quantifying type 5 metabotropic glutamate receptor (mGluR5) binding parameters with positron emission tomography (PET).

**Methods:**

We first measured mGluR5 density in postmortem human cerebellum using [^3^H]ABP688 autoradiography (n=5) and immunohistochemistry (n=6). Next, *in vivo* experiments were conducted in healthy volunteers (n=6) using a high-resolution PET scanner (HRRT) to compare [^11^C]ABP688 binding potential (BP_ND_) values obtained with reference tissue methods and the two-tissue compartment model *vs.* metabolite-corrected arterial input function.

**Results:**

The postmortem data showed that, relative to the hippocampus, the cerebellum had 35% less mGluR5 immunoreactivity and 94% fewer [^3^H]ABP688 binding sites. *In vivo* brain regional [^11^C]ABP688 BP_ND_ values using the cerebellum as a reference region were highly correlated with BP_ND_ values and distribution volumes derived by arterial input methods (R^2^ > 0.9).

**Conclusion:**

The scarce availability of cerebellar allosteric binding sites at autoradiography, compared to immunohistochemistry results, might reflect the presence of distinct mGluR5 isoforms or conformational state. Together with our PET data, these data support the proposition that [^11^C]ABP688 BP_ND_ using the cerebellum as a reference region provides accurate quantification of mGluR5 allosteric binding *in vivo*. Studies relying on this method could, therefore, be used in clinical populations, providing that stronger initial assumptions are met.

## 1 INTRODUCTION

Metabotropic glutamate receptors (mGluRs) modulate various aspects of glutamatergic neurotransmission. The eight known mGluRs are classified into three families according to their pharmacological properties [1]. mGluR5 belongs to group I, which is functionally linked to the release of intracellular calcium, activation of phospholipase C, diacylglycerol, protein kinase C, and inositol triphosphate [2].

Studies in both laboratory animals and humans provide evidence that mGluR5 is involved in dementia [3], anxiety [4], motor [5], and substance use disorders [6-10]. Moreover, converging evidence points toward a specific role for mGluR5 in molecular mechanisms of neuroprotection [11] and synaptic plasticity [12]. Given these features, quantification of receptor availability *in vivo* is crucial for testing mGluR5 hypotheses in people with neuropsychiatric disorders and evaluating novel therapeutic targets.

In contrast to most positron emission tomography (PET) receptor probes, (3-(6-methyl-pyridin-2-ylethynyl)-cyclohex-2-enone-O-11C-methyl-oxime ([^11^C]ABP688) binds with high selectivity and specificity to a transmembrane allosteric site instead of orthosteric binding sites. The *in vivo* pharmacology of imaging agents specific to allosteric binding sites is a relatively new frontier in neuroreceptor imaging since the binding pockets for allosteric activators, and inhibitors are located in the membrane compartment and do not compete directly with the transmitter [13], although it cannot be excluded that changes in neurotransmitter concentration are devoid of effects on ligand affinity [14]. Moreover, it remains unclear whether allosteric binding sites in the nervous system are exclusively available in a single conformational state.

As a PET ligand, [^11^C]ABP688 shows a favorable chemical and pharmacokinetic profile, including rapid brain uptake, high mGluR5 affinity, fast kinetics, and absence of brain permeable radiolabeled metabolites [15]. A consistent anatomical distribution pattern is seen when comparing *in vitro* and *in vivo* estimates of mGluR5-specific binding in the rodent brain [16, 17].

Most PET [^11^C]ABP688 studies have relied on a single bolus paradigm of tracer administration. A two-tissue compartmental model (2TCM) using metabolite-corrected plasma input function has been successfully employed to quantify mGluR5 availability in humans [13, 18, 19], baboons [20, 21] and rats [16, 22].

Non-invasive quantification of [^11^C]ABP688 binding parameters (*i.e*., binding potentials; BP_ND_) without arterial blood sampling has been validated in non-human species, using the cerebellum as a reference region [16, 20]. These methods are based on extremely low mGluR5 concentrations in the rodent cerebellum [19, 23]. Conducting quantification of *in vivo-in vitro* binding parameters in the rodent brain, we have found that the saturable [^3^H]ABP688 cerebellar binding sites found *in vitro* were not sensitive to competitive blocking with MPEP previously measured *in vivo* with [^11^C]ABP688 and PET [16]. However, these results cannot necessarily be translated to human studies since Patel and colleagues [24] suggest that the presence of cerebellar mGluR5 immunoreactivity and binding site availability in humans might be higher than in rodents.

Given the conflicting evidence, our goal was to conduct *in vivo* and *in vitro* experiments to test whether the cerebellum can be considered a reference region suitable for [^11^C]ABP688 quantification in humans. To that end, we performed cerebellar and hippocampal semi-quantitative mGluR5 immunohistochemistry and [^3^H]ABP688 quantitative autoradiography to characterize binding in regions previously described as harboring high and low mGluR5 densities. Additionally, we established correlations between binding parameters obtained with PET metabolite-corrected arterial input function (AIF) and the cerebellum as a reference region. Due to safety issues and Health Canada regulations, displacement studies with co-injection of large doses of [^11^C]ABP688 competitors cannot be conducted in living human individuals. Circumventing arterial cannulation would greatly simplify the procedure and limit discomfort during testing.

## 2 METHODS

### 2.1 Postmortem Brain Specimens and Ethics

Specimens were obtained from the Brain Bank from the Douglas Mental Health University Institute (Montreal, Canada). All *in vitro* experiments were carried out in accordance with the guidelines provided by the Douglas Brain Bank research board, approved by the Research and Ethics Board of the Douglas Research Institute, McGill University. Specimens utilized in this study were re-evaluated by a neuropathologist (MCG) to confirm the absence of brain pathology that could confound the results (*i.e*. cerebrovascular disease or neurodegenerative processes). The presence of ante mortem history of DSM-IV psychiatric conditions or neurological disorders was excluded based on both interviews with family members and/or treating professionals and on medical records.

### 2.2 Radiochemistry

*In vitro study:* [^3^H]ABP688 specific activity (2738 GBq/ mmol) was purchased from Amersham/GE Healthcare Biosciences (Little Chalfont, Buckinghamshire, UK). [^3^H]ABP688 was synthesized from desmethyl-ABP688 cis/ trans (1:8) produced by ABX (Radeberg, Germany) by reacting the sodium salt of desmethyl-ABP688 in anhydrous dimethyl-sulfoxide with [^3^H]methyl iodide at 90°C for 5 minutes.

*In vivo study:* Desmethyl-ABP688 cis/trans (1:8) was produced by ABX (Radeberg, Germany). [^11^C]ABP688 was synthesized by reacting the sodium salt of desmethyl-ABP688 in anhydrous dimethyl-sulfoxide with [^11^C]methyl iodide at 90°C for 5 minutes. The product was purified by semipreparative high-performance liquid chromatography (Waters, µBondapak, C18; mobile phase, acetonitrile: 0.1% phosphoric acid (30:70); flow rate, 2 mL/min), and the retention time was 10 minutes. After the removal of the high-performance liquid chromatography solvent by evaporation, the product was formulated using 9 mL of phosphate buffer and 1 mL EtOH. TLC: CH_2_Cl_2_:MeOH: AOAc (7:2:1). The radiochemical purity was > 99%. The total time required for the synthesis of [^11^C]ABP688 was 30 minutes from the end of the bombardment.

### 2.3 [^3^H]ABP688 Quantitative Autoradiography

Human frozen brain slices (n=5, demographics in Table **S1** of Supplementary Information) corresponding to the cerebellum and hippocampus regions were studied. The Hippocampal CA1 region was selected for normalized quantification across samples. Tissues were cryosectioned at 20 µm at -15°C (HM 500 M, Microm International) and thaw-mounted on poly-L-lysine pre-coated microscope slides. Brain sections were dried at room temperature for one hour and then stored in a freezer at -80°C until use. The *in vitro* [^3^H]ABP688 binding study was performed according to Hintermann *et al*. [15] with some modifications. Briefly, slides were warmed up to room temperature and pre-incubated for 20 min in buffer containing 30 mmol N_2_ HEPES, 110 nmol NaCl, 5 mmol KCl, 2.5 mmol CaCl_2,_ and 1.2 mmol MgCl_2_ (pH 7.4). A [^3^H]ABP688 saturation binding study was performed using concentrations of 8, 4, 2, 1, 0.5, 0.25, and 0.125 nM in the same buffer for 60 min at room temperature. Non-specific binding was determined with the addition of the selective, non-competitive mGluR5 antagonist 2-methyl-6-(phenylethynyl)-pyridine (MPEP, 10 µmol/L) in adjacent sections. After the incubation, slides were washed (3 × 5 minutes) in a cold buffer, dipped in ice-cold distilled water, and rapidly dried under a stream of cool air. After drying, tissues were fixed, desiccated by exposure to paraformaldehyde powder *in vacuo* overnight [25] and exposed along with [^3^H] microscales (GE Healthcare, UK) to tritium-sensitive radioluminographic imaging plates (BAS-TR, Fuji-Film, Japan) for five days. After the exposition, imaging plates (BAS-TR2025, Fuji-Film) were scanned using BAS 5000 (Fuji-Film). Imaging plates were analyzed using the software ImageGauge 4.0 (FujiFilm). Specific radioactivity was calibrated using the [^3^H] microscales and measured in regions of interest (as described below). Analysis of the saturation binding data was calculated by fitting a one-site binding model to the specific binding data using GraphPad Prism 4 Software (GraphPad Software, Inc, San Diego, USA). Values for the maximum receptor density (B_max_) and dissociation constant (K_D_) are expressed in pmol/mg of wet tissue and nmol of ligand, respectively. All statistical analyses were performed using R Statistical (version 2.10.0) and GraphPad Prism 4 software.

### 2.4 Tissue mGlu5 Receptor Immunohistochemistry

Formalin-fixed human cerebellum and hippocampus (n=6; demographics in Table **S1** of Supplementary Information) were studied. Specimens were paraffin-embedded and cut at 3 μm for mGluR5 immunohistochemistry, performed in a BenchMark XT (Ventana Medical System, Tucson, Arizona), following the manufacturer’s protocols. We developed an automatic immunohistochemistry pipeline to automatically process all slides utilized in this study, which minimizes variability across slides due to operator interaction in individual slides. Slides were incubated with a rabbit primary antibody anti-rat mGluR5 (AB5675; Millipore) and diluted at 1:100 in a blocking medium for 32 minutes at 37°C. The polyclonal antibody was generated by an in-frame insertion of 21 amino acids close to the C-terminus of the receptor. Revelation was performed with the Ventana Ultraview DAB detection kit (Ventana Medical System), followed by counterstaining. All slides underwent quality control.

### 2.5 Immunohistochemistry Analysis

Digital images from stained sections were obtained in an Aperio ScanScope AT (Aperio Technologies, Vista, California) at 20× magnification. Quantification of strong positive areas was performed with ImageScope software (Aperio Technologies, Vista, California), using its positive pixel count algorithm. Regions of interest were drawn in different regions of the sections containing the cerebellum and hippocampus, and the number of DAB positive pixels was quantified, averaged, and normalized as 100. Immunoreactivity was classified as weak (<100), moderate (between 100 and 175), and strong (>175). The final results are shown as a percentage of immunopositive pixels (number of immunopositive pixels/number of immunopositive pixels + number of immunonegative pixels).

### 2.6 *In vivo* Experiments

The study was carried out in accordance with the Declaration of Helsinki and was approved by the Research and Ethics Board of the Montreal Neurological Institute/McGill University (IRB # A09-M90-11A). Written informed consent with details of the experimental procedures and approved by our institution's Research Ethics Board was obtained from all subjects before scan sessions.

### 2.7 Subjects

Six healthy volunteers (2 women, 21-38 yo; mean age ± SD, 24.5 ± 6.6 y) participated in the [^11^C]ABP688 PET study, recruited from the community through online advertisement. After a pre-screening phone interview, potential candidates underwent a face-to-face interview with the investigator (MSM), with special attention to personal and family history of neurological and psychiatric disorders, using a semi-structured clinical interview for DSM IV (SCID). For each candidate, a medical evaluation, electrocardiogram, routine blood test (CBC, electrolytes, thyroid, cholesterol, liver, and renal workup), and urine toxicology screening were performed to assess physical health status. Volunteers were excluded in the presence of a current Axis I psychopathology or neurological disorder, a somatic disorder, and the presence and/or history of first-degree relatives with neurological or psychiatric disorders, including substance use disorders. Before a PET scan session began, all participants tested negative on a urine drug screen (Triage Panel for Drugs of Abuse, Biosite Diagnostics, San Diego, California), and women were scanned during the follicular phase and tested negative on a urine pregnancy test.

### 2.8 Positron Emission Tomography Procedures

For [^11^C]ABP688 injection, a cannula was inserted in the right arm antecubital vein. A saline solution of 362.6 ± 43.3 MBq of [^11^C]ABP688 was then administered as a 1-minute intravenous slow bolus injection, and emission scanning started concurrently with the start of the bolus injection. Participants were scanned in a supine position with a head-fixation device to minimize head movement during data acquisition. PET imaging was performed with a Siemens High-Resolution Research Tomograph (HRRT+) scanner (CTI/Siemens, Knoxville, Tennessee), which combines high spatial image resolution with high sensitivity [26]. It consists of 8 detector heads arranged in an octagon. A detector head comprises 117 detector blocks. Each is cut into 8 ´ 8 crystal elements. Each block consists of 2 lutetium oxyorthosilicate/lutetium-yttrium oxyorthosilicate (LSO/LYSO) crystal layers to achieve photon detection with depth-of-interaction information. The spatial resolution range of the scanner is between 2.3 and 3.4 mm full width at half maximum. A 7-min transmission scan was acquired before the emission scan for attenuation correction. The PET emission data were acquired in list-mode format and binned into 26-time frames. For every time frame, fully 3D sets of sinograms were generated from the list-mode data (2209 sinograms, span 9, with 256 radial bins and 288 azimuthal angle samples). A time series of 26 3D images (frames) were then reconstructed from these sinograms; each 3D image is composed of 256 × 256 × 207 cubic voxels (voxel side-length of 1.21875 mm), using an expectation-maximization image reconstruction algorithm with an ordinary Poisson model of the acquired PET data [27]. The reconstruction included full accounting for the normalization, attenuation, and time-dependent scatter and randoms. Subject head motion was corrected using a data-based motion estimation and correction method [28].

### 2.9 Arterial Blood Sampling and Metabolite Analysis

For AIF, a catheter was inserted in the radial artery by a certified anesthesiologist (MA). During the scan, 19 sequential 1 ml blood samples were manually collected. Each sample was drawn by releasing the blockade of the catheter after discarding the dead volume in the 1 mm diameter tube of a three-way stopcock. Catheters were flushed with heparinized saline solution after each sampling. For the first minute, samples were taken every 10 seconds, with increasing intervals afterward until the end of the scan (2x30 s, 3x1 min, 1x2 min, 1x3, 5x10 min). After the separation of an aliquot of whole blood, each sample was centrifuged to obtain 200 μL plasma aliquots. The concentration of radioactivity in whole blood and plasma aliquots was then measured in a gamma counter cross-calibrated with the PET system. Six additional samples of 5 mL were collected for metabolite determination at 1, 2, 5, 15, 30, and 60 minutes. The percentage of unchanged radioligand in the plasma was determined as described earlier [22]. Briefly, the lipophilic parent compound is retained by the solid phase of C18 cartridges (Sep-Pak Plus; Waters Corporation, Milford, MA, USA). The cartridges were prewashed with 5 mL of water and flushed with the plasma sample diluted in 2.5 mL of water. Hydrophilic compounds were then eluted with 5 mL of water, and the cartridge was flushed with 10 mL of air to clear the fluid. The cartridge and a 1 mL fraction of the flushing fluid were then measured in the well counter. The previous test revealed that the retention of the parent compound in a solvent to the cartridge (recovery factor) was between 91% and 96%. Therefore, the time course of the fraction of the parent compound was normalized to the first sample and was fitted by nonlinear regression analyses (*f*(t) = 1/(1 + *a* × *t*^*b*)^*c* + *d*). This fit was subsequently used to generate metabolite-corrected plasma curves.

### 2.10 Image Analysis

For anatomical co-registration, a high-resolution (1 mm) T1-weighted magnetic resonance image (MRI) acquisition was obtained for all subjects on a 1.5 T Siemens Sonata scanner, using gradient echo pulse sequence (repetition time = 9.7 msec, echo time = 4 msec, flip angle = 12°, field of view = 250 and matrix 256 × 256). Each subject’s MRI volume was manually co-registered with the 4D reconstructed PET image using the MINC tools package (McConnell Brain Imaging Center, McGill University, Montreal, Canada; http://www.bic.mni.mcgill.ca/ServicesSoftware/MINC). Regions of interest (ROIs) were manually drawn on the MRI co-registered with PET images in native space: cerebellar cortex, caudate, putamen, hippocampus, prefrontal cortex (PFC), and anterior cingulate cortex (ACC). Corresponding time-activity curves (TACs) were generated with regional activity concentration calculated for each frame, corrected for decay, and plotted *versus* time.

### 2.11 Kinetic Models

The outcome parameters measured are the equilibrium total distribution volume (V_T_) and binding potential (BP_ND_), which are directly proportional to receptor density (*B_max_*) [29]. The V_T_ is composed of the specific distribution volume (V_S_, equal to f_P_B_avail_/K_D_) and the distribution volume of free and nonspecifically bound ligands (V_ND_, which is assumed to be equivalent to the volume of distribution of the reference region). While V_T_ is dependent on blood sampling, BP_ND_ refers to a region void or with negligible specific binding and is, therefore, independent from AIF. The gray matter of the cerebellum was chosen as a reference region throughout the modeling. Kinetic analysis was performed using the iFit software package [30].

*V_T_ estimation*. A 2TCM with four unconstrained kinetic constants was applied to analyze the data. 2TCM has been previously shown to provide superior fits than the one-tissue compartment model for [^11^C]ABP688 in all brain regions, including the cerebellum [19]. Vascular contribution to tissue TACs (V_b_) was added as an additional unfixed parameter in the model. The rate constants for each ROI were determined from the TACs by non-linear curve fitting in the least-squares sense. V_T_ was defined as (K_1_/k_2_)(1 + k_3_/k_4_). To test for higher stability of the fits, a constrained 2TCM was also applied. The ratio K_1_/k_2_ in target ROIs was fixed to the value (∼10%) estimated in the reference region, under the assumption that it is similar in different brain regions.

V_T_ was also estimated using the Logan’s graphical analysis (GA) [31]. In this simplified approach, which uses plasma as an input function, V_T_ corresponds to the slope of the linear portion of the resulting plot for each ROI. The v_b_ contribution was fixed to the values derived in the 2TCM analysis. The time of linearization was set to t* = 10 minutes.

*Calculation of BP_ND_* was determined by the following methods: (1) distribution volume ratio (DVR) derived from the 2TCM (V_T_/V_ND_−1); (2) simplified reference tissue method (SRTM) [32] in which the reference region TAC is used as input function to interpret TACs of target regions through non-linear curve fitting procedure; (3) non-invasive Logan’s GA (NIGA) with a t* = 10 minutes [33]. This modified approach uses the reference region as an input function and allows for the estimation of the DVR. BP_ND_ is calculated as DVR-1. Selection of t* was based on a visual inspection of the residual plot.

### 2.12 Statistics

All values are expressed as mean ± SD. For the kinetic study, the adequacy of the fitting for each model was judged according to the Akaike information criterion (AIC), with lower AIC values indicative of a better fit. Pearson’s correlation coefficient and linear regressions were performed using SPSS (IBM SPSS Statistics, version 20.0.0) as well as ANOVA two-way analysis when needed.

## 3 RESULTS

### 3.1 *In vitro* Experiments

#### 3.1.1 Autoradiography and Saturation Binding Analysis

**Fig. (1):**
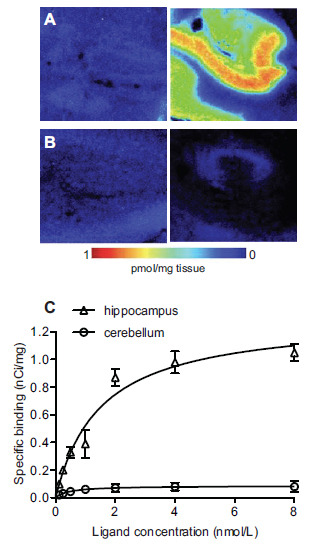
[^3^H]ABP688 binding and the effect of blockade with MPEP in the autoradiography study in human brain sections. Representative autoradiograms showing (**A**) the total binding of 2 nmol/L [^3^H]ABP688 in the cerebellum (left) and hippocampus (right), and (**B**) the nonspecific binding in the presence of the antagonist MPEP, in the cerebellum (left) and hippocampus (right). The scale shows density of mGluR5 in pmol/mg of tissue. (**C**) Saturation binding experiment with [^3^H]ABP688. Displayed are the nonlinear regression fit curves for the CA1 region of the hippocampus (high-binding region) and the cerebellum (reference region).

The mean B_max_ values were 1.31 ± 0.1 and 0.08 ± 0.01 pmol/mg of tissue for CA1 region and cerebellum (t-test, *p <* 0.001). Fig. (**1A**) shows representative total binding in the two brain areas. There was no difference in the maximum density of mGluR5 (B*_max_*) in the molecular and granular layers of the cerebellum. A hippocampus/cerebellum B_max_ ratio of 15:1 was observed. Cerebellar K_D_ was also significantly lower than hippocampal K_D_ (0.4 ± 0.02 *vs* 1.5 ± 0.2 nmol/L, t-test, *p <* 0.05). The ratio of B_max_/K_D_ in the hippocampus was 4-fold higher than in the cerebellum. Fig. (**1B**) shows representative nonspecific binding in the presence of the competitor. MPEP (2 nmol/L) reduced [^3^H]ABP688 total binding by >75% in the cerebellum (on the left) and >95% in the hippocampus (on the right) (Fig. **1C**).

#### 3.1.2 Immunohistochemistry

**Fig. (2):**
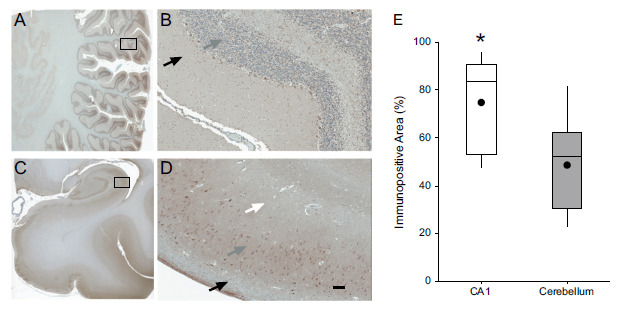
Distribution of mGluR5 in human brain sections of cerebellum (**A**, **B**) and hippocampus (**C**, **D**) at immunohistochemistry. In the cerebellum, moderate staining is observed in the molecular layer and nuclear layer (black and grey arrow in **B**, respectively). In CA1 region of the hippocampus, strong staining is observed in the soma and neuropil of the pyramidal layer (grey arrow in **D**). Lower intensity of staining is observed in the dendritic branches in the stratum radiatum (white arrow in **D**) and in the stratum oriens (black arrow in **D**). The bar in D indicates 100 micrometers. (**E**) Quantification of mGluR5-immunopositive area in cerebellum and CA1 region. The boxplots represent the lower and upper quartile and the median of the distribution, the whiskers represent the lowest and highest value found, the dots represent the means. *p < 0.001.

mGluR5 immunoreactivity was definitively positive in the human cerebellum (Figs. **2A** and **2B**). Moderate immunoreactivity was homogeneously observed throughout the molecular and granular layers of the cerebellar cortex. Purkinje cell layer was strongly immunoreactive. Neurons of the dentate nucleus showed only moderate immunoreactivity. No significant staining was observed in the white matter. In the hippocampus (Figs. **2C** and **2D**), stronger mGluR5 staining was observed in pyramidal neurons. mGluR5 staining was less pronounced in granular cells. The quantification of positive areas revealed a higher percentage of mGluR5 immunopositive area in the CA1 area of the hippocampus than in the cerebellum (Fig. **2E**, hippocampus = 74.7 ± 19.0%, cerebellum = 48.7 ± 19.2%; t-test, *p <* 0.001).

### 3.2 *In vivo* Experiments

#### 3.2.1 Regional Uptake and AIF *in vivo* PET Study

**Fig. (3):**
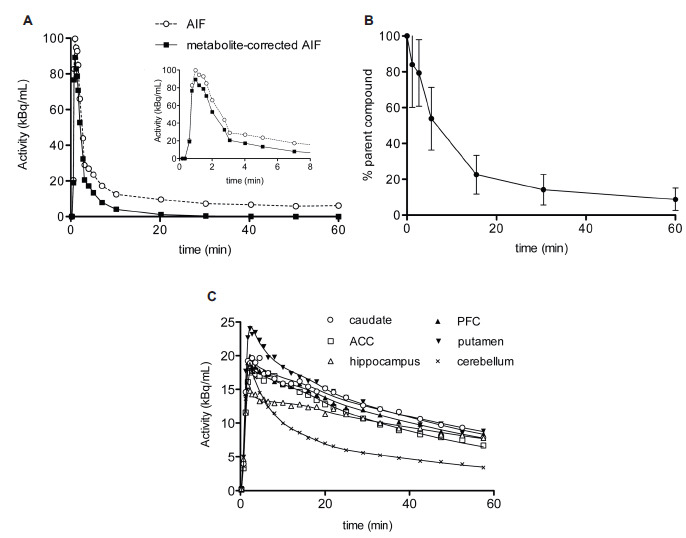
Plasma and regional time-activity curves for [^11^C]ABP688 and radiotracer metabolism. (**A**) Time-activity curves for the concentration of total radioactivity in plasma (dashed line) and of unchanged radioactivity in plasma (solid line) after correction for metabolites in a representative subject. AIF: arterial input function. The small insert shows the plasma peak in the first minutes. (**B**) Time course for the percentage of activity of parent compound in plasma (mean±S.D.; n = 6). (**C**) Representative regional time-activity curves after intravenous injection of [^11^C]ABP688 in a healthy subject. Symbols represent data for each brain region. Lines represent the fits to the data points with the unconstrained 2-tissue compartment model. **Abbreviations**: ACC: anterior cingulate cortex; PFC: prefrontal cortex.

Fig. (**3A**) shows the curves for the total concentration of radioactivity in the plasma, the metabolite-corrected plasma activity, and the fraction of the parent compound as a function of time. After injection, [^11^C]ABP688 activity in the arterial plasma reached a peak at 50-70 seconds. [^11^C]ABP688 was rapidly metabolized, with metabolites accounting for approximately 50% of the radioactivity in the plasma after 5 minutes (Fig. **3B**). The fraction of non-metabolized parent compound decreased at a slower rate thereafter, to 0.22 ± 0.1, 0.14 ± 0.8, and 0.08 ± 0.06 at 15, 30, and 60 minutes, respectively. The whole blood/plasma concentration ratio was 0.61 ± 0.12 at peak and 0.80 ± 0.12 at 60 minutes after injection. The mean specific activity of the radioligand was 0.25 ± 0.14 mCi/nmol, with a mean injected mass of 0.20 ± 0.11 μg/kg of body weight. The radioligand injected was rapidly distributed in the brain, as shown in the TACs in Fig. (**3C**). Radioactivity peaked after 1.7 minutes, and the concentration was highest in the mGluR5-rich regions, such as putamen and caudate. All other areas showed comparable levels of peak estimates, although, as expected, the cerebellum presented the fastest washout of radioactivity (more than halved at 20 minutes post-injection). [^11^C]ABP688 plasma clearance was 88.7 ± 24.3 L/h, calculated as the area under the curve and dose injected ratio.

#### 3.2.2 Kinetic Modeling Using AIF

**Fig. (4):**
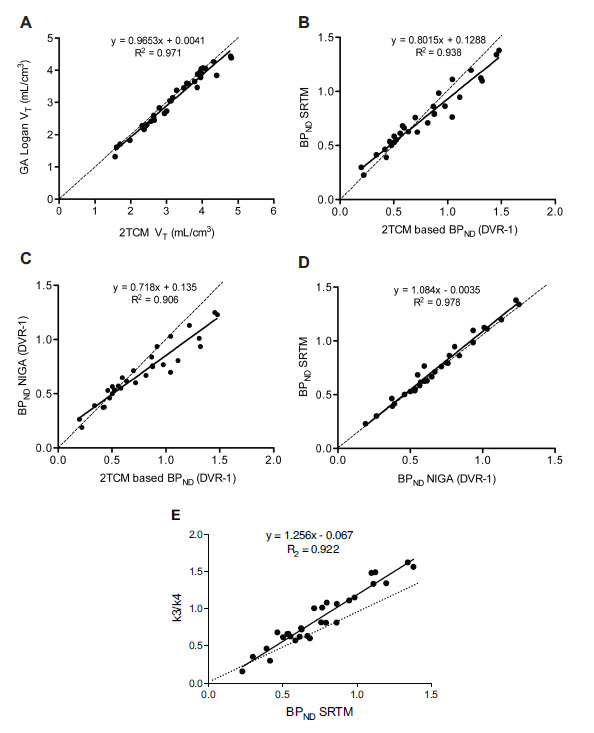
Correspondence of the outcome parameters distribution volume (V_T_) and binding potential (BP_ND_) derived by different methods of analysis. (**A**) Correlation between V_T_ values obtained by the 2-tissue compartment model (2TCM) and Logan’s graphical analysis (GA). (**B**) Correlation between BP_ND_ values derived from the V_T_ of 2TCM and the simplified reference tissue method (SRTM). (**C**) Correlation between BP_ND_ values obtained using the 2TCM and the distribution volume ratio (DVR-1) from Logan’s noninvasive GA (NIGA). (**D**) Correlation between BP_ND_ values obtained using the noninvasive reference tissue models SRTM and NIGA. (**E**) Correlation between SRTM-derived BP_ND_ and binding potentials directly derived from k3/k4 estimated with the constrained 2TCM. Data points for all brain regions from the 6 subjects are presented. The solid line represents linear regression analysis, and the dotted line represents identity. R^2^ derived from regression analysis is shown for each set, as well as the regression equation.

In the kinetic analysis, the 2TCM with unconstrained parameters adequately described all regional TACs. Representative fittings are depicted in Fig. (**3C**). Unconstrained 2TCM yielded consistently lower AIC values than those obtained by fixing K_1_/k_2_ and was therefore preferred for all subsequent analyses (values not shown). Table **1** summarizes V_T_ values derived from the 2TCM analysis. These values were estimated with high precision, as attested by a mean percent of standard error of 8.5 ± 1.8% across subjects and brain regions. The V_T_ was highest in the PFC and ACC (3.7 ± 0.7 and 3.6 ± 0.6 mL/cm^3^, respectively) and lowest in the cerebellum (∼2.0 mL/cm^3^). No correlation between V_T_ and injected mass was found. There was an excellent agreement between the V_T_ values calculated from the 2TCM and those obtained using the GA approach (*r* = 0.99, R^2^ = 0.97, *p <* 0.001; values in Table **1** and correlation graph with regression equation in Fig. **4A**).

The estimated rate constants are shown in Table **1**. K_1_ mean values were relatively uniform across all brain regions, with the highest values found in the putamen and the lowest in the hippocampus. The range of K_1_ values (0.138-0.185 mL/cm^3^/min^-1^) corresponds to a first-pass extraction of approximately 30%, considering reports of regional cerebral blood flow in humans. K_1_ standard error variability ranged from 4.4 to 8.8%. Larger but tolerable standard errors were found for k_2,_ k_3,_ and k_4_ estimations, ranging from 10-23%, 11-24%, and 8-19%, respectively. Amongst the ROIs, the 2TCM fit provided consistently stronger identification of kinetic constants in the cerebellum and putamen. V_b_ had regional values ranging between 0.08 to 0.09 ml/cm^3^.

#### 3.2.3 Comparison of BP_ND_ Measures in AIF and Reference-based Methods

BP_ND_ values derived from arterial input modeling were calculated from the V_T_ values of the target and reference regions in the 2TCM. The cerebellar cortex (grey matter) was used as a reference region in noninvasive quantifications. High correspondence between 2TCM BP_ND_ and the values derived from simplified noninvasive approaches was found (*p <* 0.001; Figs. **4B** and **4C**). In particular, SRTM provided the strongest agreement (r = 0.97, R^2^ = 0.94), although with a positive average bias of 6.4% in the putamen and a negative bias of 7.8% in the hippocampus. As expected, noninvasive methods yielded lower inter-subject variability compared to invasive models. NIGA approach provided BP_ND_ values in excellent correlation with SRTM (r = 0.99, R^2^ = 0.98, *p <* 0.001), with comparable inter-individual variability (36-40%) and a consistent underestimation of 5.0-11.0% (Fig. **4D**). The ratios of k_3_ over k_4_, with values derived from the constrained 2TCM, were in excellent agreement with BP_ND_ values from the SRTM (r = 0.96, R^2^ = 0.92, *p <* 0.001) (Fig. **4E**). Mean BP_ND_ estimates across methods are summarized in Fig. (**5**).

## 4 DISCUSSION

Our study provides two main findings supporting the use of the cerebellum as a reference tissue for deriving [^11^C]ABP688 BP_ND_ values in humans. First, the B_max_/K_D_ ratio, as measured through autoradiography, between the cerebellum and hippocampus was about 1:4, with the availability of binding sites for [^3^H]ABP688 in the cerebellum 15-fold lower than in the hippocampus. Second, there was a high correspondence between V_T_ or DVR estimated *via* 2TCM and BP_ND_ values using the cerebellum cortex as a reference region in the human PET study.

### 4.1 *In vitro* Experiments

To our knowledge, this is the first study to investigate binding parameters in the human cerebellum with [^3^H]ABP688 *in vitro* autoradiography and immunohistochemistry. Since the introduction of molecular agents for mGluR5, the cerebellum has been described as a low-uptake region when compared to regions such as the hippocampus [15, 16, 22, 34]. Consistent with these reports, we confirmed here the presence of small-magnitude cerebellar-specific binding in human subjects. The cerebellar concentration reported in our study (B_max_ of 0.08 pmol/mg tissue) is in agreement with our previous report for rats with the same techniques (B_max_ ∼ 0.09 pmol/mg tissue, with 15-fold hippocampus/cerebellum ratio) [16].

Autoradiography images (Fig. **1**) showed that the binding signal before and after competitor MPEP is similar to the background activity, supporting low cerebellar-specific binding. In our *in vitro* experimental conditions, a 10 µmol/L blocking dose of MPEP imposed 95% [^3^H]ABP688 binding reduction in the hippocampus and 75% in the cerebellum. In addition to a much smaller B_max_ compared to the hippocampus, the cerebellum shows a much lower ratio of specific to nonspecific binding. Moreover, the lower *K_D_* observed on the cerebellum suggests that the receptor’s affinity state is different from that in the hippocampus. It is improbable that the cerebellar-specific binding sites reported here represent cross-binding (*i.e*., binding to mGluR1, another group I mGluR that is abundant in the cerebellum) owing to the high ABP688 specificity to mGluR5 [15]. The low magnitude of [^3^H]ABP688 saturable binding sites, as reported here, agrees with earlier gene expression studies showing modest mGluR5 mRNA expression in the human adult cerebellum cortex [35, 36]. At least, in the case of [^3^H]ABP688, the comparable mGluR5 binding parameters between rodents and humans support the assumption that the use of the cerebellum as a reference region in the rat can be extended to humans.

Our [^3^H]ABP688 quantitative autoradiography results contrast with previous reports obtained with other mGluR5 allosteric ligands, such as [^18^F]FPEB or [^11^C]MTEP, for which the brain/cerebellum ratio was determined as a 5-fold index. These discrepancies can be attributed to primate species evaluated and methodological differences (intact *vs.* tissue homogenates) and/or method of quantification (quantitative autoradiography *vs*. “non-wash wipe” assay”) [24]. In addition, the reduced brain/cerebellum ratios previously reported for mGluR5 allosteric ligands might be in part secondary to the intrinsic pharmacokinetics properties of a given molecular probe (*i.e*., binding sites are not necessarily the same) [24, 34]. Finally, cis/trans isomerism may also play a role in the quantification of binding parameters of different mGluR5 allosteric ligands such as [^3^H]ABP688, [^18^F]FPEB, or [^11^C]MTEP. In our present study, we used a 1:8 cis/trans concentration of desmethyl-ABP688, which minimized the effects of the inactive cis-isomers [23]. To the extent that these effects were present, they would have affected absolute binding values without altering correlations between values derived using different approaches.

Interestingly, when compared to the hippocampus, we found only a 35% mGluR5 reduction in cerebellar immunoreactivity in the presence of a 15-fold reduction in the cerebellar [^3^H]ABP688 B_max_. These results are in agreement with a previous semi-quantitative study comparing mGluR5 immunoreactivity and allosteric binding site density [24] with the observation that immunohistochemistry is not a direct measurement for protein quantification. Although mGluR5 receptors are present in the cerebellum, the availability of their allosteric binding sites in comparison to mGluR5 located in the cortex and the hippocampus was markedly reduced. The biological interpretation of this dissociation is discussed below.

### 4.2 *In vivo* Experiments

TAC analysis showed a rapid brain uptake with similar levels of initial radioactivity in all ROIs and cerebellum. Consistent with previous human studies using [^11^C]ABP688 [13, 19], activity in the cerebellum decreased more rapidly than elsewhere with similar trends over time, generating TACs with robust reproducibility across studies.

Similar to our previous work in rodents, here we directly compared AIF-based models to simplified reference tissue and graphical methods using the grey matter of the cerebellum as reference tissue. In the AIF-based method, the 2TCM configuration with four unconstrained parameters fitted the data from all regions, including the cerebellum. This was expected, as previously shown for [^11^C]ABP688 in rats and humans [16, 19].

K_1_ values estimated with the 2TCM analysis showed excellent consistency within regions and between regions (ANOVA two-way, F = 1.59, *p* = 0.192), indicating uniform tracer delivery in the brain. V_T_ values in our study were similar to those reported in previous studies using an HRRT scanner [37, 38].

Interestingly, in line with reports from other tracers [39], a one-tissue compartment model does not adequately fit the cerebellum’s TAC. It is unlikely that this reflects the presence of a two-tissue compartment since the actual specific binding was shown to be minimal. More plausibly, this is due to a non-instantaneous equilibrium between free and nonspecific compartments that is usually assumed in the model simplification [40]. The model choice for the cerebellum is further supported by the significant correspondence between V_T_ 2TCM-derived and V_T_ obtained with the Logan GA (Fig. **4A**), which does not consider compartments. The slight underestimation of GA V_T_ estimates (between 2 and 4%, depending on region) is common to many different tracers and likely due to statistical noise. Also, the possible contribution to a second compartment of a small (∼5%) fraction of radioactivity due to radiolabeled metabolites that entered the brain [23] cannot be excluded.

The ratio of k_3_ over k_4_ provides a direct estimate of the binding potential that is independent of the reference region. These values, when obtained with the constrained 2TCM, were highly correlated with the SRTM-derived BP_ND_ (Fig. **4E**), providing strong evidence that the cerebellum is a suitable reference region for mGluR5 quantification.

**Fig. (5):**
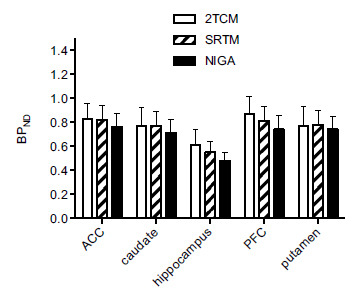
Regional binding potentials (BP_ND_) values were estimated by three different methods of analysis: metabolite-corrected plasma input 2-tissue compartment model (2TCM); simplified reference tissue method (SRTM), and noninvasive graphical analysis (NIGA). Data are presented as mean ± S.E.M. **Abbreviations**: ACC: anterior cingulate cortex; PFC: prefrontal cortex.

The data range for BP_ND_ was in line with other reports in baboons and humans [9, 18, 20, 41, 42]. Despite the high correspondence between BP_ND_ estimates across the methods used, some quantitative differences were present (Fig. **5**). SRTM did not consistently underestimate BP_ND_, as the possible presence of a second compartment would have implied [43]. However, all averaged biases with respect to 2TCM estimates were within the acceptable limit of 7%. In support of the reliability of this non-invasive method, SRTM values were also robustly correlated with those obtained by NIGA. NIGA provided the lowest binding values among all methods, independently from ROI, and a stable weak underestimation, with a pinnacle for the smallest region hippocampus (-11%), with respect to SRTM. The latter should then be considered the most valid method for measuring occupancy in future group comparison studies.

When applying reference tissue models, it is assumed that V_ND_ is homogeneous in all brain regions and equal to the reference region V_T_. Since, as shown with autoradiography, specific binding in the cerebellum is very low, one could assume that only free and nonspecifically bound radiotracers significantly contribute to cerebellum V_T_. In fact, when applying the B_max_/K_D_ ratio as an estimate of BP_ND_ values to the *in vivo* data derived from the 2TCM, V_ND_ estimation resulted in approximately 1.7, about 85% of the cerebellum V_T_.

Arterial cannulation and blood sampling are invasive procedures that require additional effort and human resources. They impose discomfort on the tested individuals, and the procedure is poorly accepted in certain clinical populations. In addition, the extraction of the metabolite-corrected AIF is one of the main sources of inter-subject variability. Therefore, non-invasive models for mGluR5 quantification with [^11^C]ABP688 using the cerebellum as a reference region are preferable and reliable.

Any condition that might have an effect on nonspecific binding measures in the cerebellum potentially invalidates the initial assumption. In particular, cerebellar atrophy can compromise the accuracy of tracer level estimations because of partial volume effects [40]. The sources of injury to the cerebellum vary, involving toxins (alcohol, chemotherapy, anticonvulsants), inflammation (autoantibodies, encephalitis), structural damage (stroke, tumors), inherited degenerations, and congenital malformations.

### 4.3 Biological Interpretation and Limitations of [^11^C]ABP688 BP_ND_

**Fig. (6):**
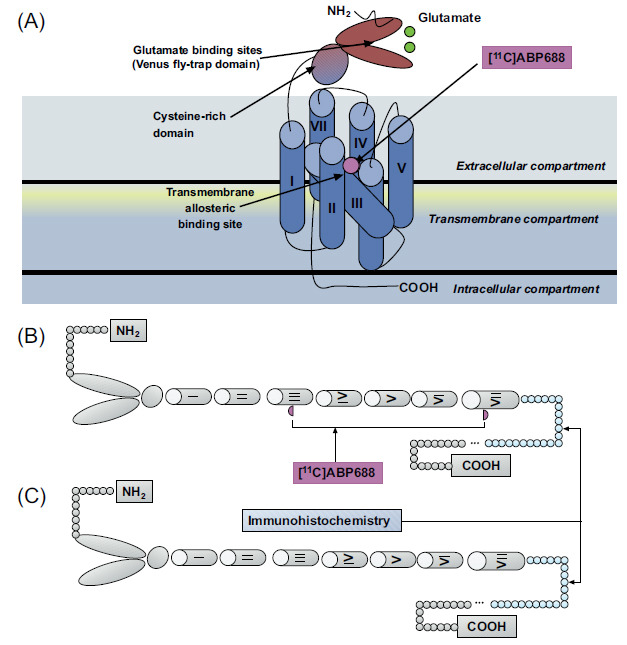
Top panel (**A**) represents mGluR5, a member of the class C family of G-protein coupled receptors displaying a large extracellular amino-terminal domain for glutamate binding (dark blue), 7 transmembrane helical segments (light blue), and an allosteric pocket (violet). The lower panel shows a schematic representation of mGluR5 with (**B**) or without allosteric binding sites available for [^11^C]ABP688 binding (**C**). The former predominates in the prosencephalon and the latter in the cerebellum. The part painted in blue refers to the amino acid sequences (AA 1189-1212) recognized by the mGluR5 polyclonal antibody used in the study.

Although there is a good agreement between metabolite-corrected plasma input function and cerebellar reference tissue methods, the present results raise some caution on the interpretation of binding parameters involving molecular probes for mGluR5 allosteric binding sites. The 15-fold hippocampus/cerebellum ratio obtained with autoradiography contrasts with the 35% difference obtained with immunohistochemistry. Although both techniques serve to quantify mGluR5, the former technique relies on the availability of the mGluR5 allosteric binding site (tertiary conformation) and the latter on a sequence of amino acids (primary conformation) [44]. This dissociation suggests that mGluR5 allosteric binding sites are unavailable in the cerebellum (Fig. **6**).

It has been proposed that the mGluR5 allosteric binding site is fundamentally a network of aromatic residues present between the 3 and 6 transmembrane loops. This binding site is highly dependent on the tertiary and quaternary receptor conformation [44]. In contrast, the mGluR5 antibody used in this study binds to a 21 amino acid sequence located in the mGluR5 C-terminus after protein denaturation and is, therefore, unaffected by receptor conformation changes [45]. Thus, reduced binding sites due to either changes in primary or tertiary mGluR5 conformation might explain these results. Possibly, [^11^C]ABP688 binding captures a specific mGluR5 population abundant in the prosencephalon and scarce in the cerebellum. Our current data showing that [^11^C]ABP688 K_D_ in the cerebellum is about 4-fold lower than in the hippocampus (0.4 *vs.* 1.5 nmol/L) implies that cerebellar receptors are in a higher affinity state. Whether this could be due to differences in glutamate concentrations is not clear, although pharmacological challenges that are thought to increase neurotransmitter levels produced changes in [^11^C]ABP688 binding [21, 46]. Interestingly, the mGluR5 splice variant most represented in the cerebellum is less sensitive to agonist-induced desensitization, with respect to other variants mostly expressed in the cerebral cortex and hippocampus [47].

These observations might also be explained by distinct cerebellar mGluR5 conformational states. Indeed, modifications in the receptor-radioligand interactions due to changes in affinity state have been described for D2 receptors, with D2 agonist PET ligands showing 2-fold displacement after amphetamine challenge compared to D2 antagonist ligands [48]. Glutamate release might reduce [^11^C]ABP688 affinity [21] as its binding to the orthosteric site mobilizes the venus fly trap domain in the receptor’s N-terminus down towards the membrane, which could alter the accessibility to the allosteric binding sites residues in the transmembrane domains and thus affect [^11^C]ABP688 affinity. However, this claim must be confirmed by further studies in which autoradiography and receptor immunodetection could be compared head to head in healthy and disease conditions.

Reference tissue methods for calculating BP_ND_ require a region with negligible specific binding of the radioligand to the receptor. Consistent with the present autoradiography data, two *in vivo* human studies using [^11^C]ABP688 and exogenous drugs have previously demonstrated some specific binding in the cerebellum [49, 50]. This may influence binding estimates using reference region approaches. Cerebellar grey matter, used in our human PET study, may be more suitable than the entire cerebellum, according to an *in vivo* competition study in baboons using a blocking agent [20]. Indeed, significant mGluR5 expression was found in the human deep cerebellar nuclei [24, 36]. Blocking studies, considered the gold standard to evaluate the suitability of a reference region, may be feasible in humans in the near future when safety concerns are overcome with new compounds. Since even a small amount of specific binding in the reference region can influence BPND estimates, arterial lines, and plasma analysis may be necessary for studies where percent occupancy or group differences are expected to be minimal.

### 4.4 Limitations of the Present Study

The external validity of the present findings requires the consideration of the following limitations. For our *in vitro* experiments, systematic comparisons between mGluR5 immunohistochemistry and binding parameters were not conducted since tissue sections used in the two techniques were derived from different sets of brains (frozen or formalin-fixed). However, it is important to emphasize that there was no age or ante mortem clinical difference between the two subject populations from which the specimens were derived. Together, our *in vitro* experiments suggest that cerebellar mGluR5 differs qualitatively and quantitatively between the cerebellum and prosencephalon. For the purpose of the present study, these data support the use of the cerebellum as reference tissue in [^11^C]ABP688 binding quantification.

Our *in vivo* experiments involved a relatively small group of healthy volunteers. In addition, it would be highly desirable to conduct these experiments with injections of the active trans-[^11^C]ABP688, although the radiochemistry used in this study results in a ratio of 1:8 [23]. Nevertheless, the isomer-related effect would only change the absolute VT/BPND values quantification without affecting the ability to examine correlations between values calculated using different methods. The use of the HRRT scanner might bias our results due to its higher sensitivity and known issues regarding scatter and attenuation correction at the level of the cerebellum. Since comparisons across multiple kinetic models were beyond the scope of the present manuscript, the kinetic analyses presented here focus on widely available methodology.

## CONCLUSION

In summary, our data on reproducibility obtained in rats showed favorable results in terms of low availability of binding sites in the cerebellum. Overall, there was excellent agreement between estimates of BP_ND_ and metabolite-corrected plasma-derived values, demonstrating the validity of models relying on reference regions to quantify mGlu5 receptor availability with [^11^C]ABP688. When changes in cerebellar cortex V_ND_ cannot be ruled out, and studies in which percent occupancy or group differences are expected to be minimal, invasive quantification methods with arterial lines are preferable.

## Figures and Tables

**Table 1:** Average kinetic parameters and total distribution volumes obtained with metabolite-corrected plasma input function.

**2TCM**					**GA Logan**	
**ROI**	**K_1_ (mL/cm^3^/min)**	**k_2_ (min^-1^)**	**k_3_ (min^-1^)**	**k_4_ (min^-1^)**	**V_T_ (mL/cm^3^)**	**V_T_ (mL/cm^3^)**
Cerebellum	0.15 ± 0.03	0.33 ± 0.14	0.08 ± 0.02	0.03 ± 0.01	2.01 ± 0.48	1.90 ± 0.48
ACC	0.14 ± 0.02	0.15 ± 0.05	0.11 ± 0.06	0.05 ± 0.02	3.60 ± 0.59	3.43 ± 0.55
Caudate	0.15 ± 0.03	0.17 ± 0.06	0.12 ± 0.06	0.04 ± 0.02	3.53 ± 0.99	3.39 ± 0.96
Hippocampus	0.14 ± 0.03	0.22 ± 0.13	0.17 ± 0.10	0.05 ± 0.01	3.17 ± 0.58	3.04 ± 0.54
PFC	0.15 ± 0.03	0.20 ± 0.11	0.13 ± 0.07	0.04 ± 0.02	3.69 ± 0.74	3.58 ± 0.70
Putamen	0.18 ± 0.02	0.22 ± 0.06	0.10 ± 0.03	0.03 ± 0.01	3.46 ± 0.64	3.39 ± 0.69

**Note**: Values are mean ± SD (*n* = 6); 2TCM, two-tissue compartment model; GA, graphical analysis; V_T_, total distribution volume; ACC, anterior cingulated cortex; PFC, prefrontal cortex.

## Data Availability

Data are available from the corresponding author upon reasonable request.

## LIST OF ABBREVIATIONS

ACC: Anterior Cingulate Cortex
AIF: Arterial Input Function
mGluRs: Metabotropic Glutamate Receptors
MRI: Magnetic Resonance Image
PET: Positron Emission Tomography
PFC: Prefrontal Cortex
TACs: Time-activity Curves
