# Supplementary material for: Quantification of [11C]ABP688 Binding to mGluR5 in Human Brain using Cerebellum as Reference Region: Biological Interpretation and Limitations
Source: Curr Neuropharmacol. 2025 Feb 18;23(9):1081–94. doi: 10.2174/1570159X23666250127161855 (PMC12272086; doi:10.2174/1570159X23666250127161855)
Supplement: Supplementary file 1 [file CN-23-9-1081_SD1.pdf]

Supplementary Material

Quantification of [^{11}C]ABP688 Binding to mGluR5 in Human Brain using Cerebellum as Reference Region: Biological Interpretation and Limitations

Michele S. Milella[1], Luciano Minuzzi[2], Chawki Benkelfat[3,4], Jean-Paul Soucy[3], Alexandre Kirlow[3], Esther Schirrmacher[5], Mark Angle[3], Jeroen A.J. Verhaeghe[6], Gassan Massarweh[3], Andrew J. Reader[7], Antonio Aliaga[3], Jose Eduardo Peixoto-Santos[8], Marie-Christine Guiot[3,9], Eliane Kobayashi[3], Pedro Rosa-Neto[3,4,5,10] and Marco Leyton[3,4,*]

[1]Toxicology Unit, Policlinico Umberto I Hospital-Sapienza, University of Rome, Italy; [2]Department of Psychiatry and Behavioural Neurosciences, McMaster University, Hamilton, ON, Canada; [3]Department of Neurology and Neurosurgery, Montreal Neurological Institute, McGill University, Montreal, Canada; [4]Department of Psychiatry, McGill University, Montreal, Canada; [5]Department of Oncology, Division of Oncological Imaging, University of Alberta, Edmonton, AB Canada; [6]Molecular Imaging Center Antwerp (MICA), University of Antwerp, Antwerp, Belgium; [7]Department of Biomedical Engineering, King's College, London, United Kingdom; [8]Department of Neurology and Neurosurgery, Paulista Medical School, UNIFESP, Sao Paulo, Brazil; [9]Department of Pathology, McGill University, Montreal, Canada; [10]Translational Neuroimaging Laboratory, Douglas Research Institute

1875-6190/25 $65.00+.00 **© 2025 Bentham Science Publishers**

Table S1. Demographics from donors of tissue specimens used in the present study. There were no group differences (AR *vs*. IHC) in age (*p*=0.9; t=0.09; df=9) or postmortem processing time to freezing/fixation (*p*=0.2; t=1.38; df=9).

Specimen	Experiment	Age	Sex	Postmortem Delay (Hours)	Side	Cause of Death/Diagnosis
1	AR	76	M	20.5	R	Larynx neoplasia
2	AR	82	F	32.5	R	Acute Myocardial Infarct
3	AR	78	F	19.7	R	Renal failure
4	AR	48	F	43.7	L	Lung neoplasia
5	AR	77	F	17.2	L	Acute Myocardial Infarct
6	IHC	95	F	23.75	L	Hepatic Metastasis
7	IHC	85	M	26.75	L	Lung Carcinoid Tumor
8	IHC	88	M	8	L	Ruptured abdominal aortic aneurism
9	IHC	51	F	26.25	L	Metastatic lung cancer
10	IHC	61	M	8.75	L	Pancreatic neoplasm
11	IHC	59	M	17.67	L	Gastric neoplasm

Legends: AR (autoradiography), IHC (immunohistochemistry), F (female), M (male), R (right), L (left).
